# Peach Kernel Extracts Inhibit Lipopolysaccharide-Induced Activation of HSC-T6 Hepatic Stellate Cells

**DOI:** 10.1155/2022/4869973

**Published:** 2022-08-29

**Authors:** Hong-Jie Chen, Jin-Yuan Huang, Chih-Yuan Ko

**Affiliations:** ^1^Department of Clinical Nutrition, People's Hospital of Leshan, Leshan 614000, China; ^2^Department of Clinical Nutrition, Suzhou Dushu Lake Hospital, Suzhou 215123, Jiangsu, China; ^3^Department of Clinical Nutrition, The Second Affiliated Hospital of Fujian Medical University, Quanzhou 362000, China; ^4^School of Public Health, Fujian Medical University, Fuzhou 350122, Fujian, China

## Abstract

There is an important role for hepatic stellate cells (HSCs) in liver fibrosis. As it stands, many traditional Chinese medicine formulations can effectively improve liver fibrosis, whether it is clinically used or in animal studies; however, the efficacy and mechanism of the main formulations remain unclear, including the peach kernel, which contains numerous phytochemicals with a wide range of biological activities. The purpose of this study was to investigate peach kernel's anti-liver fibrosis effects. In this study, peach kernel extracts inhibited lipopolysaccharide (LPS) activation in HSC-T6 cells and the expression of *α*-smooth muscle actin and connective tissue growth factor induced by LPS in HSC-T6 cells. Furthermore, peach kernel extracts inhibited signal transducers involving protein kinase B and mitogen-activated protein kinase, which regulate downstream genes associated with inflammation. As a result, peach kernel extracts inhibited inflammatory responses and subsequently inhibited LPS-induced transformation of activated HSC-T6 cells.

## 1. Introduction

Cirrhosis is the result of chronic alcoholism, nonalcoholic steatohepatitis, or viral hepatitis. In the process of cirrhosis, the liver tissue is replaced by the fibrotic scar tissue to regenerate nodules, resulting in loss of the liver function [1]. Sustained hepatocyte injury induces the release of cytokines and growth factors such as transforming growth factor-*β* (TGF-*β*), tumor necrosis factor-*α*, or lipopolysaccharide (LPS), which activate hepatic stellate cells (HSCs) in the process of liver fibrosis. Myofibroblasts transformed from quiescent HSCs into activated myofibroblasts deposit more *α*-smooth muscle actin (*α*-SMA) and more extracellular matrix. It is these processes that play a vital role in the development and emergence of liver fibrosis [1, 2]. The activation of nuclear factor kappa B (NF-*κ*B) is a central link between liver injury, fibrosis, and hepatocellular carcinoma (HCC) [3–5]; however, liver fibrosis and HCC may be prevented or treated by inhibiting their activation.

When liver fibrosis progresses, patients may develop liver dysfunction and portal hypertension. Several treatment options are available currently for liver fibrosis (both in clinical trial settings and in clinical settings), which are grouped into several categories based on their mechanisms of action, such as candesartan combined with ramipril for eradication of hepatitis C virus infections or a combination of entecavir, fuzheng huayu, and traditional Chinese medicine (TCM) granules, to eliminate hepatitis B virus. In autoimmune hepatitis, anti-inflammatory drugs (corticosteroids) are used. In alcoholic hepatitis, antioxidants (candesartan and ursodeoxycholic acid) can be used. In nonalcoholic hepatitis, aldafermin, an analog of FGF19, inhibits glycogenesis to increase insulin sensitivity. Besides being anti-inflammatory (treatment for nonalcoholic hepatitis with obeticholic acid inhibits NF-*κ*B activation), it also inhibits stellate cell activation (interferon-*γ* or colchicine) [6, 7]. Studies have shown that Chinese herbal formulas can treat liver fibrosis, examples include turtle shell decoction [8], Yinchenhao decoction [9], Xiayuxue decoction [10, 11], and Yiguanjian decoction [12, 13]. On HSCs or carbon tetrachloride (CCl4)-induced liver fibrosis in rats, these Chinese formulas have shown good curative effects. Nevertheless, these formulas are all mixed with Chinese herbal medicines, making it difficult to determine which one is the active ingredient.

TCM classifies cirrhosis under the categories of fiber accumulation in the body, ascites, and jaundice. The peach kernel is derived from the dried mature seeds of the plant *Prunus persica (L.) Batsch* or the mountain peach *Prunus davidiana (Carr.) Franch*. A common use of peach kernel is to promote blood circulation and remove blood stasis and is often cited as an ingredient of TCM (Xiayuxue decoction), used for treating liver fibrosis. However, there are still no clear studies regarding its antifibrosis effect. This study aimed to investigate the antifibrosis effect of peach kernels and to explore the target of the peach kernel in anti-liver fibrosis using LPS-induced fibrosis of the HSC-T6 hepatic stellate cell line.

## 2. Materials and Methods

### 2.1. Preparation of Peach Kernel Extracts

The scientific Chinese medicine peach kernels (Sun Ten Pharmaceutical Co., Ltd., Taiwan) were added to methanol to a concentration of 10 mg/mL and placed at 4°C for 24 h after sonicating for 15 min. Ultrasonic vibration was performed again for 15 min, after which it was subjected to 70°C for 30 min, and then, the supernatant was obtained by centrifugation at 14,000 g for 10 min for subsequent experiments.

### 2.2. Cell Culture

The HSC-T6 cell line was obtained from Millipore (#SCC069, MA, USA), and the cell culture conditions were based on the previous study [14]. In brief, cells were grown at 37°C in Dulbecco's minimum essential medium (DMEM; Gibco, NY, USA) containing 2% fetal bovine serum and antibiotics involving 100 *μ*g/mL streptavidin and 100 U/mL penicillin in a humidified environment with 5% CO_2_.

### 2.3. Cell Viability Assay

HSC-T6 (2 × 10^4^) cells were cultured in 24-well plates in triplicate and added with indicated concentrations of peach kernel extracts and stimulated with 100 ng/mL LPS extracted with *Escherichia coli* O111 : B4 (Sigma, St. Louis, MO, USA) for 24–72 h. Twenty microliters of WST-1 reagent (Sigma) was added to the culture medium. The supernatant was transferred to the 96-well plate after 4–6 hours at 37°C. With EIA Reader, absorbance values at 450 nm (650 nm as a reference value) were determined.

### 2.4. Western Blot

HSC-T6 (5 × 10^5^) cells were cultured overnight in a 6 cm dish. The cells were treated with indicated concentrations of peach kernel extracts and stimulated with 100 ng/mL LPS for 24 h, and then, the cell extract was harvested with RIPA-containing protease inhibitors (GE Healthcare Bio-Sciences) and phosphatase inhibitor (1 mM NaF and 1 mM Na_3_VO_4_). Alternatively, cells were treated with different concentrations of peach kernel extract for 24 h. The cell culture medium was replaced and cultured in a serum-free medium for 4 h and then treated with 100 ng/mL LPS for 30 minutes, and cell extracts were collected for subsequent experiments. Protein concentration in cell extracts was detected with Bio-Rad Protein Assay Dye reagent. Protein (20–50 *μ*g) was subjected to 12% SDS-PAGE gel and separated by electrophoresis and was subsequently transferred to a PVDF membrane using a transfer device. The membrane was incubated in a blocking buffer containing anti-Smad2 (Cell Signaling Technology Inc., Beverly, MA, USA), anti-phospho-smad2 (Cell Signaling), anti-*α*-SMA (ABclonal, MA, USA), anti-connective tissue growth factor (CTGF; ABclonal), anti-protein kinase B (AKT; Cell Signaling), anti-phospho-AKT (Cell Signaling), anti-p42/p44 extracellular signal-regulated kinase (ERK; Cell Signaling), anti-phospho-ERK (Cell Signaling), anti-c-Jun N-terminal kinase (JNK; Cell Signaling), anti-phospho-JNK (Cell Signaling), anti-p38 (Cell Signaling), anti-phospho-p38 (Cell Signaling), or anti-actin (Sigma) at 4°C overnight. After three washes of 10 min each in PBS containing 0.1% Tween 20, the membranes were then placed with horseradish peroxidase-conjugated secondary antibodies for 2 h at 25°C and analyzed using an ECL kit (Amersham, Bucks, UK) to measure the protein expression.

### 2.5. Immunofluorescence Staining

HSC-T6 (1 × 10^5^) cells were seeded on slides containing poly-l-lysine coating and placed in a 6-well dish for overnight culture. Cells were treated with indicated concentrations of peach kernel extracts for 24 h. The cell medium was replaced with serum-free DMEM for 4 h, stimulated with 100 ng/mL LPS for 30 minutes, and then fixed with PBS containing 4% paraformaldehyde at 25°C for 2 h. Then, cells were perforated with PBS containing 0.1% Triton X-100 and incubated in a blocked buffer (PBS containing 2% BSA) for 1 h. Cells were stained with anti-Smad antibody followed by Alexa Fluor 488 anti-mouse IgG antibody (Invitrogen) for 1 h. Nuclei were stained with DAPI reagent for 15 minutes; then, slides were embedded with a mounting reagent and observed under a fluorescence microscope (Leica TCS SP5 scanner, Bensheim, Germany).

### 2.6. Statistical Analysis

Data were obtained from triplicate samples of each independent biological sample and presented as mean ± standard deviation. The method of statistical analysis was verified by Student's *t*-test. The difference was considered statistically significant when the *p* value was less than 0.05.

## 3. Results

### 3.1. Peach Kernel Extracts Inhibit LPS-Induced Proliferation of HSC-T6 Cells

**Figure 1:**
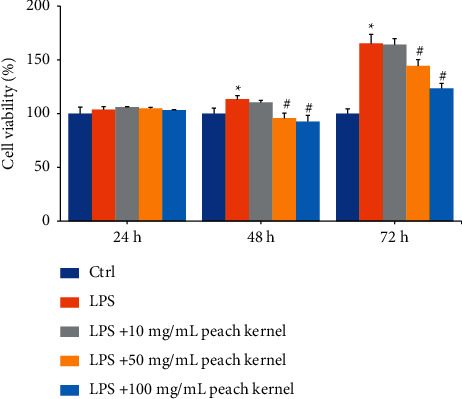
Peach kernel extracts inhibit LPS-induced cell growth of HSC-T6 cells. HSC-T6 cells were treated with indicated doses of peach kernel extract and 100 ng/mL LPS for 24–72 h. Cell viability was detected with WST-1 reagent. ^*∗*^*p* < 0.05 compared with control group; ^#^*p* < 0.05 cells were treated with LPS and peach kernel extracts compared to cells were treated with LPS.

In the course of liver fibrosis, reactive oxides activate HSC-T6 and promote cell proliferation. Thus, we first tested whether peach kernel extracts affected the LPS-induced proliferation of HSC-T6 cells.  Figure 1 shows that HSC-T6 is stimulated by LPS at 100 ng/mL as in the previous study [15]. In comparison with the control group, cell viability increased significantly at 48–72 h. Peach kernel extracts showed dose-dependent inhibition of the LPS-induced increase in cell viability in cells treated simultaneously with the extract (64 to 153% inhibition in 48 to 72 hours).

### 3.2. Peach Kernel Extracts Inhibited LPS-Induced HSC-T6 Cell Activation

**Figure 2:**
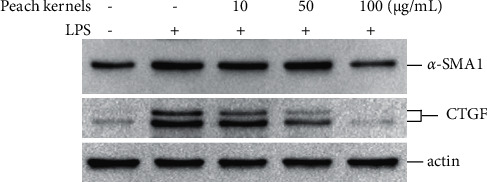
Peach kernel extracts inhibit HSC-T6 cell activation induced by LPS. HSC-T6 was treated with indicated doses of peach kernel extract and LPS for 24 h. Cell lysates were harvested, and the protein level of *α*-SMA and CTGF was detected by western blotting.

HSC-T6 activation is mediated by CTGF and *α*-SMA [16]. In this study, peach kernel extracts were tested further to determine whether they could affect LPS-stimulated CTGF and *α*-SMA production of HSC-T6. After treatment with LPS for 24 h, both *α*-SMA and CTGF levels of HSC-T6 cells increased, suggesting activation of these cells. With the addition of peach kernel extracts, the level of *α*-SMA and CTGF was significantly lower, especially at high doses (100 *μ*g/mL) (Figure 2). Based on these results, the peach kernel extract can inhibit the activation of HSC-T6.

### 3.3. Peach Kernel Extracts Inhibit LPS-Induced Smad Phosphorylation and Nuclear Translocation

**Figure 3:**
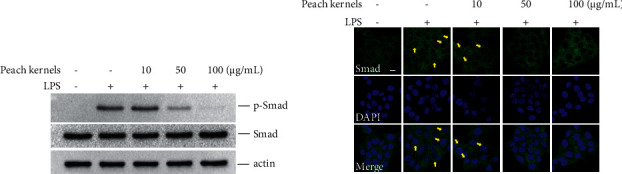
Peach kernel extracts inhibit LPS-induced Smad phosphorylation and nuclear translocation. HSC-T6 cells were treated with indicated doses of peach kernel extracts for 24 h. Cells were starved for 4 h and then stimulated with LPS for 30 min. Protein level of p-Smad and total Smad was examined by western blotting. The localization of Smad was detected by immunofluorescent. Arrows indicate Smad nuclear staining. Scale bars = 10 *μ*m.

Smad signaling is activated by both LPS and TGF-*β* in the liver and contributes to hepatic fibrosis [15, 17, 18]. Our next step was to investigate the effects of peach kernel extracts on LPS-activated HSC-T6-mediated Smad signaling. Upon stimulation with LPS, p-Smad was expressed intracellularly. The peach kernel extract inhibited the amount of p-Smad in cells in a dose-dependent manner (Figure 3(a)). When phosphorylated, Smad protein entered the nucleus where it functioned as a transcription factor that induces the expression of downstream genes, such as *α*-SMA and fibronectin. In addition, we observed the effect of peach kernel extracts on the localization of Smad protein within the cells. Figure 3(b) shows that LPS stimulation can lead to the entry of Smad into the nucleus, and with the increase in the dose of peach kernel extracts, Smad entry into the nucleus gradually decreases.

### 3.4. Peach Kernel Extracts Inhibits LPS-Induced Phosphorylation of AKT and Mitogen-Activated Protein Kinase (MAPK) Proteins

**Figure 4:**
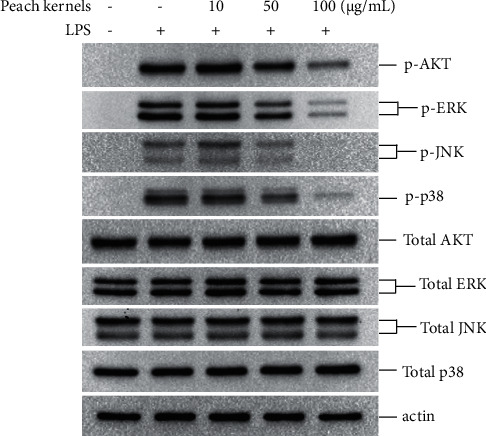
Peach kernels extracts inhibit LPS-induced phosphorylation of AKT and MAPK. HSC-T6 cells were treated with indicated doses of peach kernel extracts for 24 h. Cells were starved for 4 h and then stimulated with LPS for 30 min. The level of indicated protein was detected by western blotting.

Smad is mainly activated by LPS through the MAPK or AKT pathways [15]. We, therefore, explored which pathway is involved in LPS-regulated Smad activation by peach kernel extracts. As shown in Figure 4, LPS can cause phosphorylation of AKT, ERK, p-38, or JNK. As the dose of peach kernel extracts increased, the phosphorylation of any of the above MAPK or AKT was significantly inhibited (Figure 4). These results suggested that peach kernel extracts inhibited the activation of Smad by inhibiting the activation of downstream proteins of LPS.

## 4. Discussion

Peach kernels are used in TCM to prevent blood stasis, promote blood circulation, and moisten the intestines and laxatives. It is commonly used in the treatment of amenorrhea, dysmenorrhea, sputum, tumbling injuries, internal dryness, and constipation. Peach kernels and turtle shells are often considered magic treatments for liver cirrhosis but their role in the liver is still unclear. In this study, peach kernel extracts inhibit the downstream signaling of LPS and LPS-induced activation of HSC-T6 cells was inhibited, suggesting that in addition to promoting blood circulation and alleviating blood stasis, the peach kernel could also inhibit inflammation and inhibit the activation of HSCs, thereby alleviating liver fibrosis. As a result of this study, Xiayuxue decoction was partially found to be effective at inhibiting liver fibrosis in *in vivo* animal experiments [11].

In fact, several studies have confirmed that peach kernel ingredients are effective bioactives [19, 20]. Polyphenols and tetraterpenoids are the main active components of the peach kernel. Polyphenols act as antioxidants and protect the body against cancer, aging, cardiovascular disease, and diabetes [21, 22]. The tetraterpenoids are polyenes composed of 40 carbons. It has antiatherosclerosis, anti-inflammatory, and immunomodulatory properties similar to polyphenols, making it useful in the treatment of age-related degenerative diseases, cancer, cardiovascular disease, and macular degeneration [23]. Efficacy of polyphenols in liver disease prevention and treatment has been widely introduced [24, 25]. By inhibiting the NF-*κ*B activity, polyphenols can reduce liver inflammation and treat nonalcoholic fatty liver disease [26, 27]. It can also increase *β*-fatty acid oxidation [28], inhibiting lipogenesis through MAPK activation, downregulating sterol regulatory element-binding protein-1c [28, 29], and enhancing antioxidant defense through nuclear factor erythroid 2-related factor 2 (Nrf2) pathway [30]. Tetraterpenoids may also prove effective in treating liver disease [31, 32]. Tetraterpenoids may inhibit the development of liver disease by inhibiting NF-*κ*B activation and by activating the Nrf2 pathway [33]. Nevertheless, polyphenols and tetraterpenoids have been reported to have many targets for the prevention or treatment of liver diseases; peach kernels contain numerous compounds of polyphenols and tetraterpenoids that have anti-inflammatory properties. The biological activity of each unit cannot be excluded, and it is difficult to examine each one individually.

Peach kernels are rarely used alone in clinical practice. Xiayuxue decoction, for instance, contains 9 grams of rhubarb, 9 grams of peach kernels, and 9 grams of a soft-shelled turtle. Gavage at 2.5–10 g/kg for 4 weeks significantly reduced CCl_4_-increased alanine aminotransferase (ALT) and aspartate aminotransferase (AST) levels in a rodent experiment [11]. In a human study, taking Ja-Wei-Ge-Xia-Zhu-Yu-Tang (72 grams of the TCM) containing 9 grams of peach kernels daily for 24 weeks improved biochemical test values (ALT and AST levels) in patients with liver cirrhosis [34]. It implies that more clinical trials are needed for these TCM compounds with different afflicted subjects to prove their efficacy in preventing or treating liver diseases. In contrast to oral administration, peach kernels are mainly used externally, alongside plant oil products that protect and enhance the skin's immune system [35, 36]. Since peach kernel oil is credited with smoothing and moisturizing the skin and improving elasticity, many peach kernel oil products are available for use on the skin. Depending on the origin and preparation process, peach kernels may differ in effectiveness when used internally as TCM. However, recent research and development of TCM have utilized many different standards for interpreting peach kernel data [37, 38], which can reduce the divergence in drug activities produced in different regions, resulting in the future promotion of a wide range of scientific TCM.

## 5. Conclusions

In this study, peach kernel extracts inhibited the activation of HSCs by LPS. Peach kernel extracts inhibited the expression of *α*-SMA, a marker of cellular fibrosis, and the activation of its regulator, Smad. Furthermore, we investigated the impact of peach kernel extract on LPS downstream regulators AKT and MAPK, which suggests that the peach kernel extract mainly inhibited HSC-T6 cell activation by inhibiting the inflammatory response.

## Data Availability

The data used to support the findings of this study have been included in this article.
